# Variability of respiratory rate measurements in neonates- every minute counts

**DOI:** 10.1186/s12887-021-03087-z

**Published:** 2022-01-03

**Authors:** Catherine Muthoni Njeru, J. Mark Ansermino, William M. Macharia, Dustin T. Dunsmuir

**Affiliations:** 1 The Aga Khan University Hospital, 3rd Parklands Avenue, Nairobi, Kenya; 2 The University of British Columbia, 4480 Oak Street, Vancouver, British Columbia Canada

**Keywords:** Neonates, Respiratory rate measurement, Variability

## Abstract

**Background:**

Respiratory rate is difficult to measure, especially in neonates who have an irregular breathing pattern. The World Health Organisation recommends a one-minute count, but there is limited data to support this length of observation. We sought to evaluate agreement between the respiratory rate (RR) derived from capnography in neonates, over 15 s, 30 s, 120 s and 300 s, against the recommended 60 s.

**Methods:**

Neonates at two hospitals in Nairobi were recruited and had capnograph waveforms recorded using the Masimo Rad 97. A single high quality 5 min epoch was randomly chosen from each subject. For each selected epoch, the mean RR was calculated using a breath-detection algorithm applied to the waveform. The RR in the first 60 s was compared to the mean RR measured over the first 15 s, 30 s, 120 s, full 300 s, and last 60 s. We calculated bias and limits of agreement for each comparison and used Bland-Altman plots for visual comparisons.

**Results:**

A total of 306 capnographs were analysed from individual subjects. The subjects had a median gestation age of 39 weeks with slightly more females (52.3%) than males (47.7%). The majority of the population were term neonates (70.1%) with 39 (12.8%) having a primary respiratory pathology. There was poor agreement between all the comparisons based on the limits of agreement [confidence interval], ranging between 11.9 [− 6.79 to 6.23] breaths per minute in the one versus 2 min comparison, and 34.7 [− 17.59 to 20.53] breaths per minute in the first versus last minute comparison. Worsening agreement was observed in plots with higher RRs.

**Conclusions:**

Neonates have high variability of RR, even over a short period of time. A slight degradation in the agreement is noted over periods shorter than 1 min. However, this is smaller than observations done 3 min apart in the same subject. Longer periods of observation also reduce agreement. For device developers, precise synchronization is needed when comparing devices to reduce the impact of RR variation. For clinicians, where possible, continuous or repeated monitoring of neonates would be preferable to one time RR measurements.

## Background

Respiratory rate (RR), though regarded as critically important for the diagnosis and management of respiratory and non–respiratory disease, remains a difficult parameter to monitor reliably [1]. It is affected by various factors including fever, feeding, agitation as well as sleep versus awake state [2]. This challenge is amplified in neonates, who have an irregular breathing pattern during the first few months of life as the respiratory control mechanisms mature [3]. The current recommendation from the World Health Organisation (WHO) is to count breaths over 60 s [4]. This one-time measurement is then used to decide if an infant has respiratory disease and to direct clinical management decisions. The Integrated Management of Newborn and Childhood Illnesses booklet, for example, gives the criteria of more than 60 breaths/minute in an infant less than 2 months old, to classify the child as having a possible severe bacterial infection or very serious disease [5].

Counting the rate for shorter periods and then multiplying by a factor to estimate a one-minute rate is commonly utilized but has been suggested to amplify observer error. On the other hand, counting for a longer period of time may be impractical in a busy clinical environment. Given the high variability of the breathing pattern in neonates, there is uncertainty on whether a 60 s measurement accurately captures a rate that is reflective of the respiratory status. Indeed, there is no proven method or period that is considered a gold standard for monitoring the respiratory rate [6]. Capnography was the method of choice in monitoring RR since previous studies have shown that exhaled CO2 offers an accurate and reliable means of measuring respiratory frequency [7, 8]. We were also able to analyse the raw waveform to ensure we captured the precise timing of each and every breath. A better understanding of the impact of the duration of monitoring will guide clinicians on the utility of RR while making clinical decisions.

We therefore sought to evaluate agreement between the recommended 60 s compared to 15 s, 30 s, 120 s and 300 s of respiratory rate derived from capnography in neonates.

## Methods

Following ethics approval and informed consent, neonates (age < 28 days) were enrolled in the Evaluation of Technologies for Neonates in Africa (ETNA) study. The primary purpose of the ETNA platform was to establish the accuracy and feasibility of emerging continuous multiparameter monitoring devices, the EarlySense (EarlySense Ltd., Israel) an under-mattress device and the Sibel Advanced Neonatal Epidermal (ANNE) device (Sibel Inc., Evanston, IL, USA), in measuring vital signs in neonates when compared with verified reference devices [9]. The study was conducted at the neonatal unit of Aga Khan University Hospital and at Pumwani Maternity Hospital, both in Nairobi, Kenya. Information on gestational age, gender, current weight, anthropometric measurements, clinical signs, and diagnosis were collected for all neonates. This present study was performed following the initial verification of the reference device. This verification phase was used to investigate the factors, such as physiological respiratory rate variability, that might impact the accuracy of respiratory rate measurement.

The Masimo Rad-97 pulse CO-oximeter with capnography (Masimo Corporation, USA) was used to collect continuous capnograph waveforms from neonates. Following data collection, the capnograph (carbon dioxide, CO_2_) waveform data at approximately 20 Hz was inputted into a custom breath detection algorithm developed in MATLAB (Math Works, USA) based on adaptive pulse segmentation [10], which was originally validated using ventilated patient data from capnobase.org [11] but has also been validated using visual counting in neonates. The algorithm analysed the waveform’s shape and identified the start and end of each breath (waveform trough to trough), and this breath duration was used to calculate an instantaneous respiratory rate (breaths per minute) for each breath. A mean respiratory rate was calculated by taking the mean of the instantaneous rates for all breaths within the epoch. A breath was considered to be within the epoch if its peak, as identified by the algorithm, was within the epoch. Additionally, the algorithm calculated a capnography quality score at 2 Hz. The statistical program R was used to process this information and randomly select high quality 5-min epochs for each subject. The high-quality criteria threshold was 90% of RR quality scores in the epoch meeting the minimum quality score of two, indicating a regular capnography waveform with an appropriate shape. The quality score range is from 0 to 6 as a summation of 0–1 for up-slope quality, 0–1 for down-slope quality, 0–1 for time interval regularity, and 0–3 for normality and consistency of waveform shape.

For each selected 5-min epoch, the mean RR of the first 60 s was compared to the derived mean RR measured over the first 15 s, 30 s, 120 s, full 300 s, and last 60 s (beginning 3 min later). The agreement was calculated using Bland Altman analysis [12] that compares two quantitative measurements by plotting the differences against the average and calculating the bias and 95% limits of agreement (LOA). The bias denotes the average of the mean differences between the two sets of values, while the LOA represents the interval that contains 95% of the differences between them. The bias and LOA from each comparison were then displayed on plots. We noted that variability seemed significantly increased at higher RRs. To account for this, we took the data through normalization by dividing the bias and the limits of agreement by the overall mean and expressing this as a percentage. Intra-patient agreement was assessed by comparing the first and last 1 min of the five-minute period.

## Results

A total of 327 capnograph waveforms from 327 subjects were available from the ETNA data set, collected between June 2019 and December 2020. For 21 cases, we were not able to select a full 5-min epoch of high-quality data, and so these cases were excluded. As per the study protocol [8], measurements were recorded for at least 1 h and the median length of recorded capnography across the 306 cases was 4:20:23. As each round of analysis of the original ETNA study used a sample size of 200 for between-device comparisons [9], our 306 sample size that included one sample from each case was deemed sufficient for this analysis. The diagnoses of the remaining 306 neonates included a wide range of conditions typical of the new-born unit (Table 1). The population included both pre-term and full-term neonates with 39 (12.8%) having a primary respiratory pathology.

**Table 1:** Neonate Characteristics

Characteristic (units)	Median (range)	Median (range) RR over first 1 min
Gestational Age **in weeks (**(weeks)	39 (25–44)	53 (21–122)
25–28 weeks gestation (extremely preterm)	1%	69 (43–71)
28–32 weeks gestation (very preterm)	3%	58 (36–92)
32–37 weeks gestation (moderate to late preterm)	26%	54 (29–97)
37 weeks and above (term)	70%	51 (21–122)
Weight **in grams** (grams)	2970 (885–5075)	
Apgar Score at 5 min	9 (2–10)	
Oxygen Saturation (%)	98 (91–100)	
**Characteristic**	**n (%)**	
Sex – Males	146 (47.7)	53 (21–122)
Females	159 (51.9)	52 (24–100)
Other	1 (0.4)	48
Primary Diagnosis		
Asphyxia	35 (11.4)	
Hypoglycemia	8 (2.6)	
Jaundice	31 (10.1)	
Low birth weight	11 (3.6)	
Meconium aspiration syndrome	11 (3.6)	
Healthy neonate	69 (22.5)	
Other primary diagnosis	15 (4.8)	
Sepsis/suspected sepsis	32 (10.5)	
Prematurity	66 (21.6)	
Respiratory distress syndrome	28 (9.2)	

**Fig. 1:**
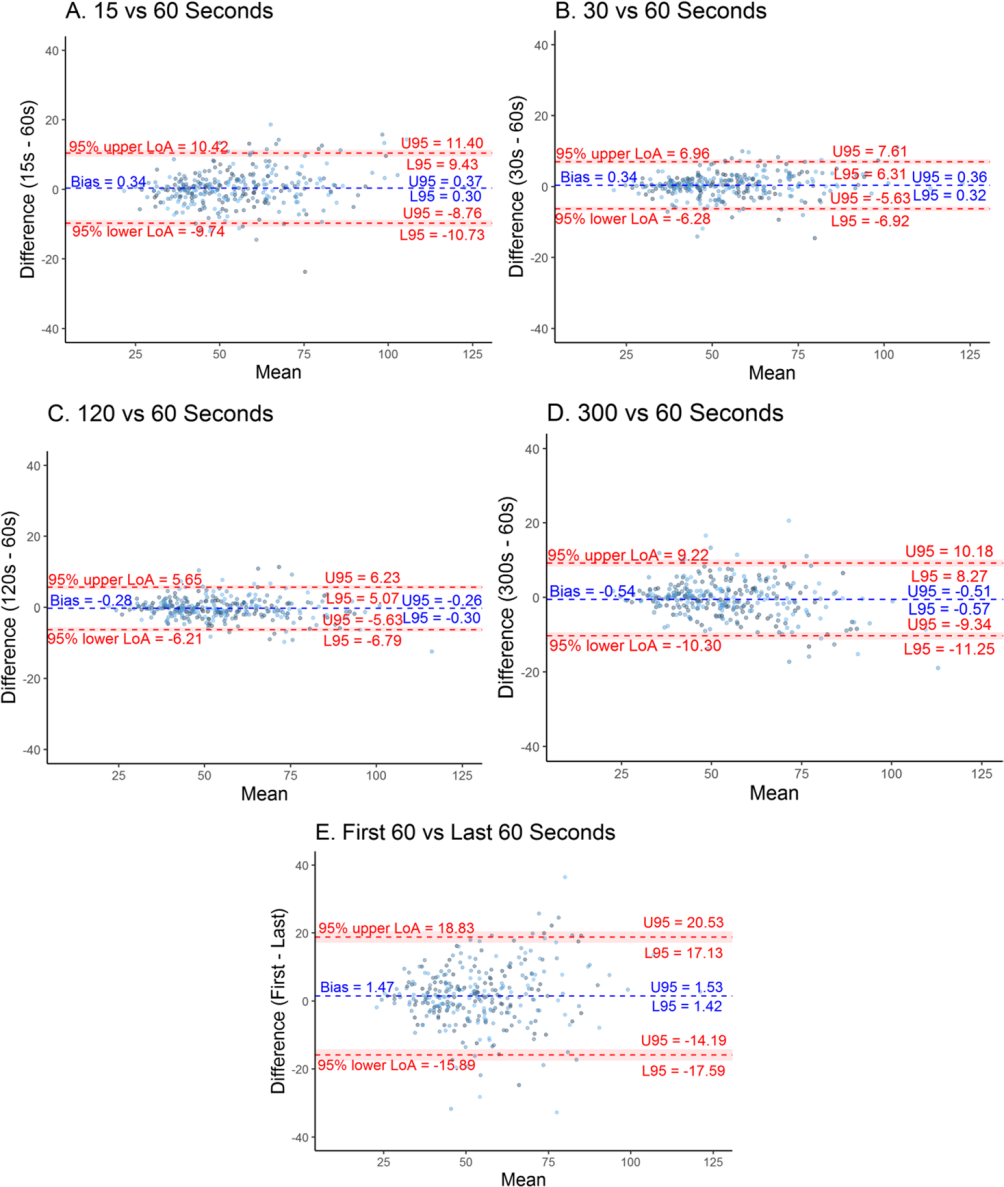
Bland Altman Plots (A to E) comparing RR values of different time periods

**Fig. 2:**
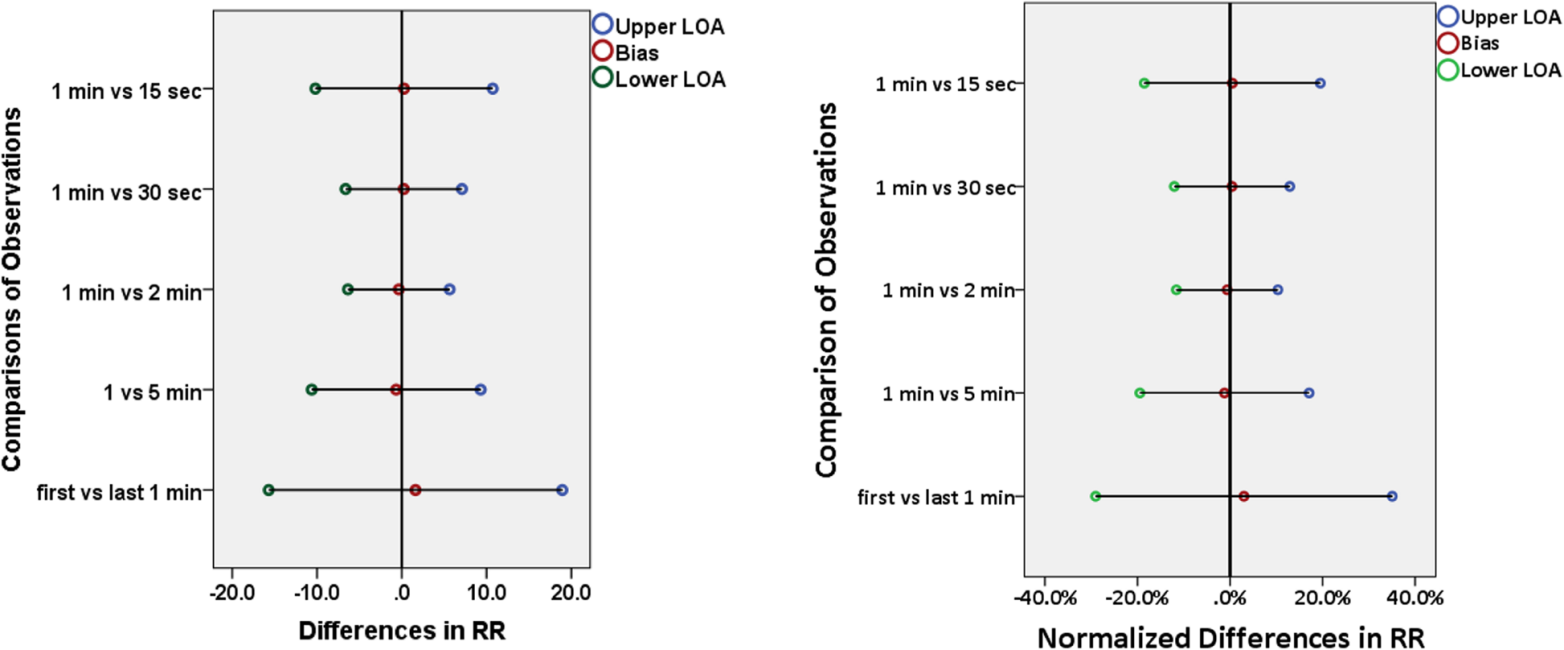
Plot showing the bias and spread of limits of agreement from different durations of observations with normalized RR values expressed as percentages on the right

There was poor agreement between all the comparisons, with a large spread of the 95% LOA [CI], ranging from 11.9 [−6.79 to 6.23] breaths per minute in the one versus 2 min comparison, to 34.7 [−17.59 to 20.53] breaths per minute in the first versus last minute comparison (Fig. 1). The LOA were increased with both shorter and longer periods of observation, but the largest LOA range was with the repeated observations in the same subject, showing significant intra-patient variability (Fig. 2).

## Discussion

The findings of this study demonstrate that respiratory rate in neonates varies considerably over a 5-min period. Furthermore, measuring respiratory rate for longer than 1 min had poor agreement with the standard one-minute rate. Measuring the respiratory rate over 15 and 30 s also reduced agreement with the one-minute rate but this was a relatively small effect compared to within individual variability. These findings provide important information for device developers looking to validate new methods and techniques for measuring respiratory rate and for clinicians measuring respiratory rate in neonates.

When comparing two independent measurements, lack of agreement may be due to the lack of precision of timings of the measurements but is also significantly degraded by the within subject variability in respiratory rate. To accurately compare two methods of respiratory rate measurement, the synchronization in time must be very precise – the same exact time period (and breaths) should be used. Furthermore, depending on a regular inter-breath duration, as is used by some devices, to derive the respiratory rate, may not account for the highly variable respiratory pattern of neonates.

For clinicians, this study demonstrates that the respiratory rate in neonates is highly variable, even over a short period of time, and so a single one-minute measurement may not accurately depict the true physiological status of the patient. We observed that the limits of agreement (LOA) was widest when comparing a one-minute period to a second one-minute period only 3 min later. From the start to the end of a 5-min period the RR of a neonate can increase or decrease by 15 breaths per minute. Continuous or repeated RR monitoring is therefore likely to be more beneficial than a single spot check measurement.

The optimum time period for counting the respiratory rate is yet to be determined. We found in this study that measuring RR over periods of more than 1 min did not improve agreement. As the measurement period increased, the agreement to one-minute decreased. There was a difference of 6 breaths/minute within the spread of 95% LOA when comparing two-minutes with the standard one-minute rate, and a difference of 10 breaths/minute with the 5-min comparison. Increasing the duration of respiratory rate counting for longer than 1 min will likely not improve accuracy. To aid in monitoring of respiratory rate, it may be best for respiratory rate monitors to display measures or visuals of the variability of the patient’s respiratory rate, such as a histogram of the last 5 min.

Measuring RR over periods of less than one-minute also showed reduced agreement, with the 15 s to one- minute comparison, for example, seeing the spread of LOA of 10 breaths per minute. Yet, this is a relatively small effect compared to the within individual variability that was demonstrated by comparing two one-minute RR counts, taken 3 min apart.

The 30 s period has previously been found to be imprecise since errors are multiplied when converting to “per minute” rates [4]. In this study, our method of respiratory rate measurement (using the mean of the instantaneous (single breath) rates of all included breaths) does not include this typical multiplication – there is no increased imprecision in measurements under 1 min, only that they are calculated based on less breaths. Despite this difference, in this study we still found the 30 s to one-minute comparison displayed wide variability with a spread of 7 breaths per minute falling within the 95% LOA.

In a similar study done in children under 5 years of age, comparing different counting periods to a synchronous capnograph recording, they found the least variability when two - 30 s measurements, done 3 min apart, were used [13]. This difference in findings is possibly due to the different time periods used for comparison (30 versus 60 s). Moreover, our study was exclusively in neonates who have higher variability in their breathing compared to older children. It important to consider this difference between neonates and older children when determining the frequency of RR measurements.

Hypoxia and increased work of breathing have been found to be more important than tachypnoea and auscultatory findings in diagnosing childhood pneumonia [14]. A 2019 commentary suggests using a combination of signs and symptoms and biomarkers (for example, C reactive protein levels) in making a clinical diagnosis of pneumonia [15].

More studies need to be done to investigate the optimum time period for evaluating respirations in the newborn and the benefit of repeat or continuous assessments in making clinical decisions.

### Limitations of the study

Respiratory rate variability may be affected by agitation, hypoxia, sleep versus awake state, and fever [16–18]. A previous study, for example, suggested a RR correction factor of 7–11 breaths per minute for each one degree °C elevation in temperatures above 38.5 degrees [19]. Sleep state has also been shown to have a marked effect on the cardio-respiratory system with irregularities being more common in active sleep compared to quiet sleep [17]. These factors were not adjusted for during our analysis. We also did not adjust for other physiological factors such as temperature, time of day, age, or gestational age that may contribute to the variability in respiration.

Furthermore, this was a cohort of mostly healthy newborns so the findings may not be generalizable to very sick neonates. We also used only high quality capnograph readings, potentially eliminating subjects who had a higher variability of respiratory rates.

## Conclusion

Neonates have high variability of RR, even over a short period of time. Variability is observed to increase with rising RR. In addition, measuring RR for shorter periods may reduce agreement with the standard one-minute rate, though this may be a relatively small effect compared to intra-patient variability. Longer periods of observation also reduce agreement. For device developers, precise synchronization is needed when comparing devices. For clinicians, where possible, continuous or repeated monitoring of neonates would be preferable to one time RR measurements.

## Data Availability

The dataset supporting the conclusions of this article is available in the Dyrad repository, 10.5061/dryad.jdfn2z3c0
